# Supplementary material for: Mice use robust and common strategies to discriminate natural scenes
Source: Sci Rep. 2018 Jan 22;8:1379. doi: 10.1038/s41598-017-19108-w (PMC5778028; doi:10.1038/s41598-017-19108-w)
Supplement: Supplementary file 1 — Supplementary information [file 41598_2017_19108_MOESM1_ESM.pdf]

Supplementary information

Mice use robust and common strategies to discriminate natural scenes

Yiyi Yu†[,1], Riichiro Hira†[,1], Jeffrey N. Stirman[1], Waylin Yu[2], Ikuko T. Smith[2], Spencer L. Smith*[,1,3,4]

† *Co-first authors*

[1] Neuroscience Center, [2] Department of Pharmacology, [3] Department of Cell Biology and Physiology, [4] Carolina Institute for Developmental Disabilities, University of North Carolina - Chapel Hill, North Carolina 27599, USA

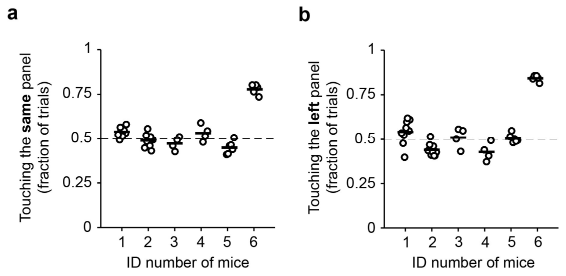

Supplementary figure 1. History dependency and choice bias

(**a**) Mouse number 6 tended to select the same side of the touchscreen on subsequent trials, while other mice did not. (**b**) Mouse number 6 tended to select the left panel, while the other mice were more even in their selections.

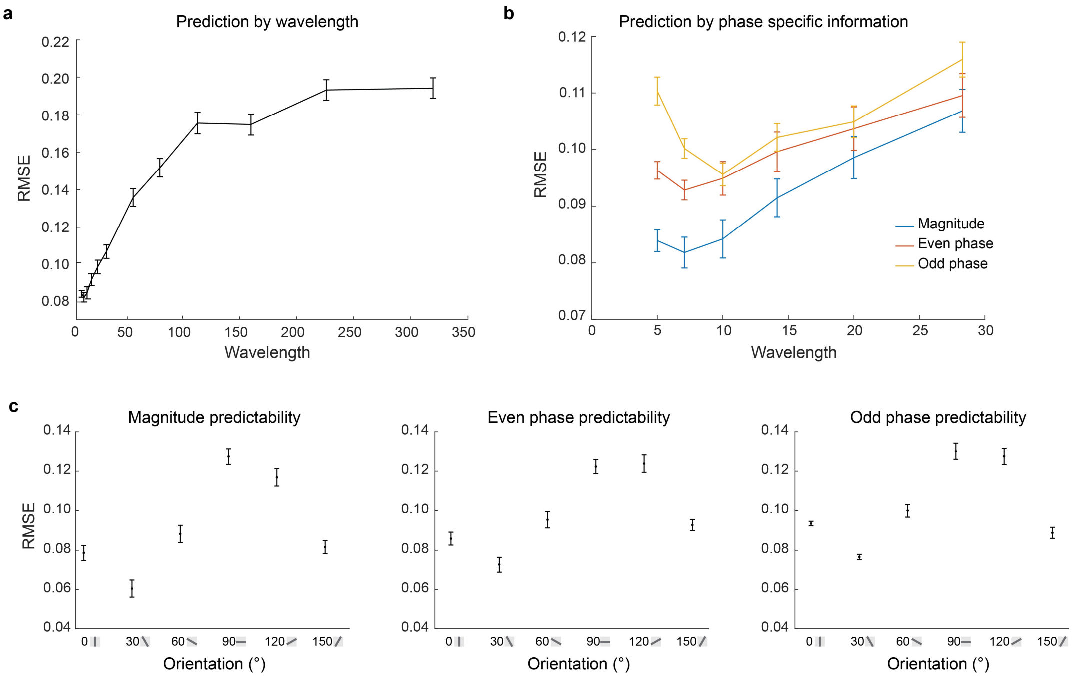

Supplementary figure 2. Gabor model analysis.

(**a**) The prediction accuracy is reducing using Gabor filter with increasingly larger wavelength. (**b**) The prediction accuracy calculated by magnitude response of Gabor filter and odd phase and even phase response of Gabor filter over a range of wavelength. (**c**) The orientation bias of Magnitude response of Gabor filter (*left*), and even phase response (*middle*) and odd phase response (*right*).

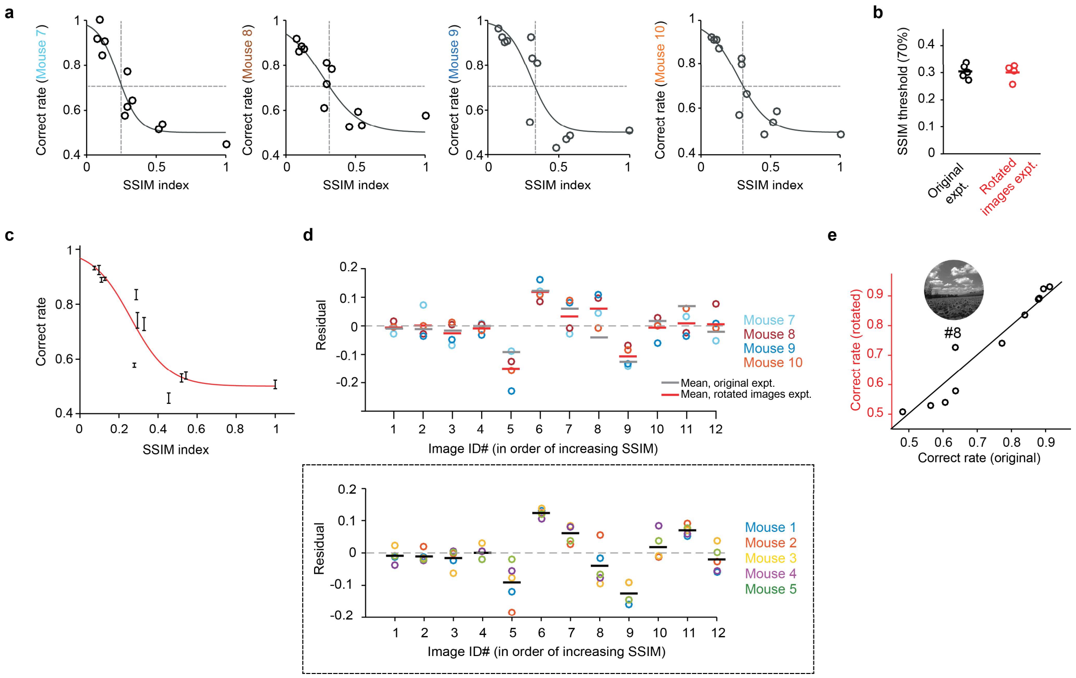

Supplementary figure 3. The mice in the rotated images experiment also displayed high consistency.

(**a**) The correct rate of each mouse in additional experiments (with rotated images) were plotted against the SSIM indices. (**b**) From those curves, the threshold SSIM values were computed (*red*) and compared to those from the main experiment (*black*). (**c**) The mean (± SEM) correct rates of mice in the rotated images experiments were plotted as a function of SSIM index (*red line* is a psychometric curve fit for the mean data). (**d**) The residual analysis in main experiments (*bottom*) and additional experiments (*top*). The mean correct rate of main experiments in each distractor image was overlaid in the top figure (*gray bars*). (**e**) The mean correct rates for each distractor image of the rotated image experiments were plotted against those of main experiments. The most deviated distractor image was shown (ID #8). Together this analysis shows that mouse-to-mouse correct rates were highly consistent, even across the main experiments and those with rotate images.

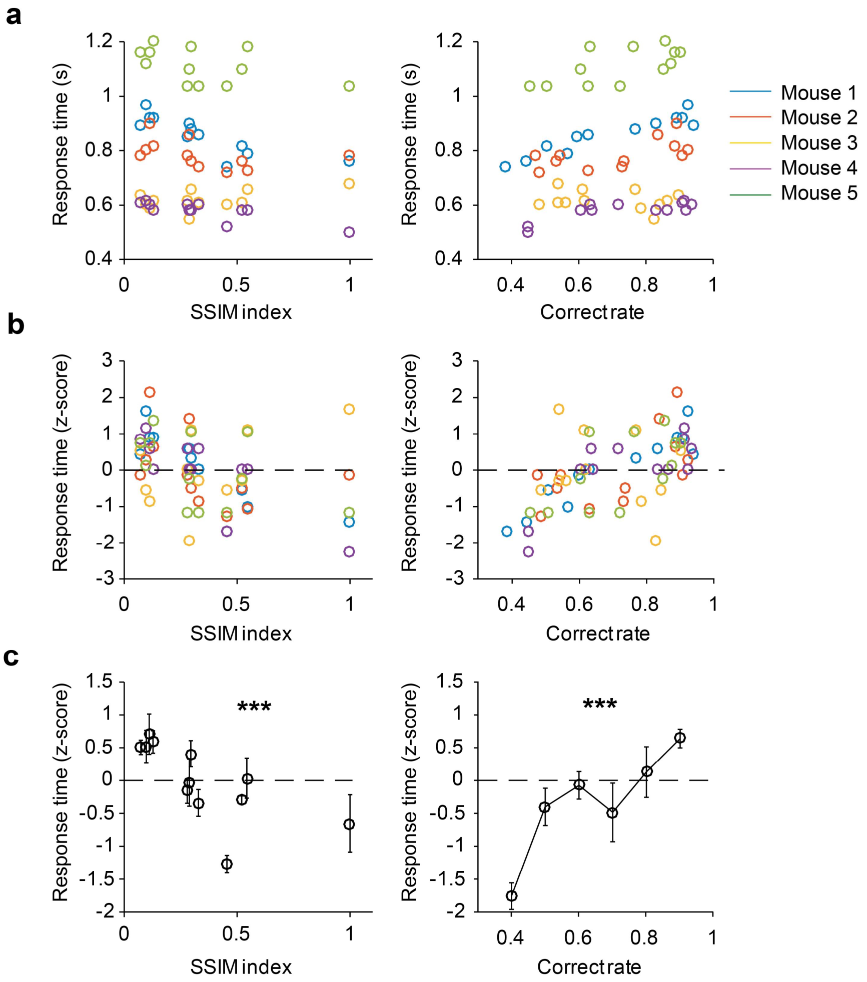

Supplementary figure 4. Response time (RT) vs SSIM.

(**a**) RT were plotted as a function of SSIM indices (*left*) and performance (correct trial rate, *right*), and data points are color-coded for each mouse. (**b**) Raw RTs from panel a were Z-scored for each mouse, to aid in mouse-to-mouse comparisons. (**c**) Mean and SEM (across all mice) for RT vs. SSIM index and RT vs. correct rate.

(Left panel, c = -0.43; right panel, c = 0.61; ***p<0.001, Spearman's rank correlation test, two-tailed).

Image ID	Image presented in the experiment	Image type	Image used (block)	SSIM	Data base	URL (file name)
0	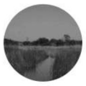	Target	Training, testing, interleaved training	N/A	Upenn	http://tofu.psych.upenn.edu/~upennidb/gallery2/main.php?g2_itemId=6254
1	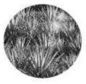	Distractor	Testing	0.074	McGill	http://tabby.vision.mcgill.ca/html/Jpgpic/Foliage/samplemerry_mexico0186.jpg
2	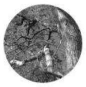	Distractor	Testing	0.098	Upenn	http://tofu.psych.upenn.edu/~upennidb/gallery2/main.php?g2_itemId=315
3	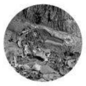	Distractor	Testing	0.11	McGill	http://tabby.vision.mcgill.ca/html/Jpgpic/Foliage/samplepippin_park0034.jpg
4	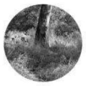	Distractor	Testing, interleaved training	0.14	Upenn	http://tofu.psych.upenn.edu/~upennidb/gallery2/main.php?g2_itemId=240
5	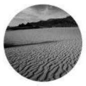	Distractor	Testing	0.28	MIT	http://cvcl.mit.edu/scenedatabase/opencountry.zip (des16.jpg)
6	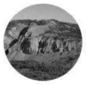	Distractor	Testing	0.29	MIT	http://cvcl.mit.edu/scenedatabase/opencountry.zip (n18053.jpg)
7	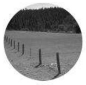	Distractor	Testing	0.29	MIT	http://cvcl.mit.edu/scenedatabase/opencountry.zip (fie33.jpg)
8	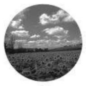	Distractor	Testing	0.33	MIT	http://cvcl.mit.edu/scenedatabase/opencountry.zip (land500.jpg)
9	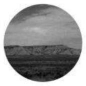	Distractor	Testing	0.45	MIT	http://cvcl.mit.edu/scenedatabase/opencountry.zip (land957.jpg)
10	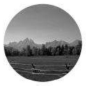	Distractor	Testing	0.48	MIT	http://cvcl.mit.edu/scenedatabase/opencountry.zip (fie35.jpg)
11	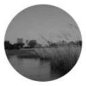	Distractor	Testing	0.54	Upenn	http://tofu.psych.upenn.edu/~upennidb/gallery2/main.php?g2_itemId=6215
12	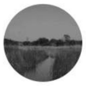	Distractor	Testing	1	Upenn	http://tofu.psych.upenn.edu/~upennidb/gallery2/main.php?g2_itemId=6254

Supplementary table 1. Images used during NID testing phase

The target image and all distractor images for NID testing block and interleaved training block are listed. The SSIM index and information for the database for each image are also listed.

Image ID	Image presented in the experiment	Image type	Image used (block)	SSIM	Data base	URL
0	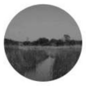	Target	Training, testing, interleaved training	N/A	Upenn	http://tofu.psych.upenn.edu/~upennidb/gallery2/main.php?g2_itemId=6254
13	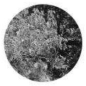	Distractor	Training	0.082	Upenn	http://tofu.psych.upenn.edu/~upennidb/gallery2/main.php?g2_itemId=5929
14	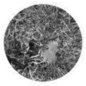	Distractor	Training	0.085	McGill	http://tabby.vision.mcgill.ca/html/Jpgpic/Foliage/sampleMerry_0068_Lasalle.jpg
15	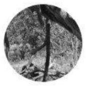	Distractor	Training	0.097	Upenn	http://tofu.psych.upenn.edu/~upennidb/gallery2/main.php?g2_itemId=5851
16	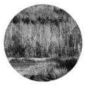	Distractor	Training	0.1	McGill	http://tabby.vision.mcgill.ca/html/Jpgpic/LandWater/samplemerry_mtl07_050.jpg
17	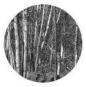	Distractor	Training	0.11	McGill	http://tabby.vision.mcgill.ca/html/Jpgpic/Foliage/samplemerry_mtl07_051.jpg
18	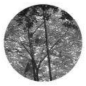	Distractor	Training	0.12	McGill	http://tabby.vision.mcgill.ca/html/Jpgpic/Foliage/samplepippin_Peel015.jpg
19	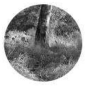	Distractor	Training	0.13	Upenn	http://tofu.psych.upenn.edu/~upennidb/gallery2/main.php?g2_itemId=240
20	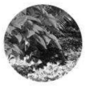	Distractor	Training	0.14	McGill	http://tabby.vision.mcgill.ca/html/Jpgpic/Foliage/samplemerry_florida0028.jpg
21	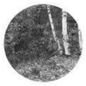	Distractor	Training	0.14	McGill	http://tabby.vision.mcgill.ca/html/Jpgpic/Foliage/samplepippin0255.jpg
22	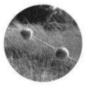	Distractor	Training	0.16	Upenn	http://tofu.psych.upenn.edu/~upennidb/gallery2/main.php?g2_itemId=16409

Supplementary table 2. Images used during NID training phase

A target image and all distractor images for training phase are listed. The SSIM index and information for the database for each image are also listed.

Supplementary video 1. A task performing mouse.

A mouse performing the NID task, during the training phase. The mouse receives a reward when it selects (i.e., touches) the target image, avoiding the distractor. Selecting the distractor image results in a time-out period.
